# Intraoperative radiotherapy in elderly patients with breast cancer: long-term follow-up results of the prospective phase II trial TARGIT-E

**DOI:** 10.1186/s12885-025-15289-0

**Published:** 2025-12-04

**Authors:** Elena Sperk, Claire Lemanski, Christian Neumaier, Juliane Lauer, Victor Siefert, Ragna Völker, Christoph Lindner, Peter Niehoff, Agnes Richard Tallet, Séverine Racadot, Adeline Petit, Montserrat Pazos, Magali Le-Blanc Onfroy, Etienne Martin, Christiane Reuter, Robert Michael Hermann, Fabian Pohl, Kay Friedrichs, Florian Würschmidt, Steffi Pigorsch, Hans-Christian Kolberg, Heiko Graf, Thomas B. Brunner, Wolfram Malter, Tjoung-Wong Park-Simon, Cosima Brucker, Jochen Fleckenstein, Cordula Petersen, Benjamin Tuschy, Eva Ekas Wilken, Sylvia Büttner, Marc Sütterlin, Henrik Flyger, Frederik Wenz

**Affiliations:** 1 Department of Radiation Oncology, Medical Faculty Mannheim, University Medical Center Mannheim, Heidelberg University, Theodor-Kutzer-Ufer 1-3, 68167 Mannheim, Germany; 2 Mannheim Cancer Center, Medical Faculty Mannheim, University Medical Center Mannheim, Heidelberg University, Theodor-Kutzer-Ufer 1-3, 68167 Mannheim, Germany; 3 Institut de Recherche en Cancérologie de Montpellier (U1194), Department of Radiation Oncology, Montpellier Cancer Institute (ICM), University of Montpellier - Campus Val d’Aurelle, 208 Avenue Des Apothicaires, 34298 Montpellier, France; 4 Clinical Center Worms, MVZ Strahlentherapie Worms GmbH, Gabriel-Von-Seidl-Str. 81, 67550 Worms, Germany; 5 Department of Anaesthesiology, Intensive Care Unit and Emergency Medicine, Hospital Ludwigshafen, Bremserstraße 79, 67063 Ludwigshafen, Germany; 6 Brustzentrum City, Alexianer St.-Gertrauden Krankenhaus, Paretzer Str. 12, 10713 Berlin, Germany; 7 Agaplesion Diakonieklinikum Hamburg, Hohe Weide 17, 20259 Hamburg, Germany; 8 Department of Radiation Oncology, Kliniken Köln, Universität Witten/Herdecke, 51067 Neufelder Str. 34Köln, Germany; 9 Department of Radiation Oncology, Sana Klinkum Offenbach, Starkenburgring 66, 63069 Offenbach, Germany; 10 Radiotherapy Department, Insitut Paoli Calmettes, 232 Boulevard de Sainte-Marguerite, 13009 Marseille, France; 11 Radiotherapy Department, Centre Léon Bérard, 28 Rue Laënnec, 69008 Lyon, France; 12 Department of Radiation Oncology, Institut Bergonié, 229 Cr de l’Argonne, 33076 Bordeaux, France; 13 LMU Klinikum, Klinik und Poliklinik für Strahlentherapie und Radioonkologie, Campus Innenstadt, Ziemssenstr. 1 80, 336 München, Germany; 14 Institut de Cancérologie de l’Ouest (ICO), Boulevard Jacques Monod, 44805 Saint Herblain, France; 15 Georges-François Leclerc Center, Department of Radiotherapy, 1 Rue du Pr Marion, BP77980, 21079 Dijon, France; 16 Radiologie Plus, Kantonsspital Frauenfeld, Pfaffenholzstrasse 4, 8501 Frauenfeld, Switzerland; 17 Center for Radiotherapy and Radiooncology Bremen and Westerstede, An der Hössen 34, 26655 Westerstede, Germany; 18 Department of Radiotherapy, Hannover Medical School, Carl-Neuberg-Straße 1, 30625 Hannover, Germany; 19 Department for Radiotherapy, Regensburg University Hospital, Franz-Josef-Strauß-Allee 11, 93053 Regensburg, Germany; 20 Mammazentrum HH am Krankenhaus Jerusalem, Moorkamp 2–6, 20357 Hamburg, Germany; 21 Radiologische Allianz Hamburg, Mörkenstraße 47, Hamburg, 22767 Germany; 22 Technical University of Munich, School of Medicine and Health, Department of RadioOncology and Radiotherapy, Ismaningerstrasse 22, 1675 München, Germany; 23 Department of Gynecology and Obstetrics, Marienhospital Bottrop, Josef-Albers-Str. 70, 46236 Bottrop, Germany; 24 Südthüringer Brustzentrum am Helios Klinikum Meiningen, Bergstraße 3, 98617 Meiningen, Germany; 25 Department of Radiation Oncology, University Medical Center Magdeburg, Leipziger Str. 44, 39120 Magdeburg, Germany; 26 Medizinische Universität Graz, Auenbruggerplatz 32, 8036 Graz, Austria; 27 University of Cologne, Medical Faculty and University Clinic of Cologne, Center for Integrated Oncology Aachen Bonn Cologne Düsseldorf, Department of Gynecology and Gynecologic Oncology, Kerpener Str. 62, 50937 Köln, Germany; 28 Department of Obstetrics and Gynaecology, Hannover Medical School, Carl-Neuberg-Str. 1, 30625 Hannover, Germany; 29 University Women’s Hospital, Paracelsus Medical University Nuremberg, Prof. Ernst Nathan-Str. 1, 90419 Nürnberg, Germany; 30 Department of Radiotherapy and Radiation Oncology, Saarland University Medical Center, Kirrberger Str. 100, 66421 Homburg, Germany; 31 Department of Radiotherapy, Westpfalz-Klinikum Kaiserslautern, Hellmut-Hartert-Str. 1, 67655 Kaiserslautern, Germany; 32 Department of Radiotherapy and Radiation Oncology, Medical Center Hamburg-Eppendorf, Medical Faculty University of Hamburg, Martinistrasse 52, 20246 Hamburg, Germany; 33 Department of Gynecology and Obstetrics, University Medical Center Mannheim, Medical Faculty Mannheim, Heidelberg University, Theodor-Kutzer-Ufer 1–3, 68167 Mannheim, Germany; 34 Department of Oncology Herlev and Gentofte Hospital, University of Copenhagen, Borgmester Ib Juuls Vej 1, 2730 Herlev, Denmark; 35 Department of Medical Statistics, Biomathematics and Information Processing, University Medical Center Mannheim, Medical Faculty Mannheim, Heidelberg University, Theodor-Kutzer-Ufer 1–3, 68167 Mannheim, Germany; 36 Department of Gynecology and Obstetrics, University Medical Center Mannheim, Medical Faculty Mannheim, Heidelberg University, Theodor-Kutzer-Ufer 1–3, 68167 Mannheim, Germany; 37 Department of Breast Surgery, Herlev and Gentofte Hospital, University of Copenhagen, Gentofte Hospitalsvej 3B, 2900 Hellerup, Denmark; 38 Medical Faculty, University of Freiburg, Hugstetter Str. 55, 79106 Freiburg im Breisgau, Germany

**Keywords:** Breast cancer, Accelerated partial breast irradiation (APBI), Intraoperative radiotherapy (IORT), Breast-conserving surgery, Toxicity, Cosmetic outcome, Elderly patients, TARGIT

## Abstract

**Background:**

Whole-breast radiotherapy (WBRT) after breast-conserving surgery (BCS) in older patients can be challenging due to the increased presence of comorbidities, comedication, the presence of a pacemaker or difficulties in traveling to treatment every day. Challenging times, such as the pandemic, can also lead to RT not being performed despite the indication. Very short treatment regimens are therefor of special interest in this population reducing overall treatment time and radiation exposure. TARGIT-E is a phase II trial investigating intraoperative radiotherapy (IORT) during BCS in elderly patients. We report long-term follow-up results of TARGIT-E.

**Methods:**

Patients with BC (≥ 70 years, cT1-2, cN0, M0) were enrolled at 28 European centers. A single dose of IORT (20 Gy) was given during BCS. Additional postoperative WBRT was applied if risk factors were present in final histopathology. Primary outcome was local recurrence-free rate (RFR) using the Kaplan–Meier-method. Late toxicities were assessed by LENT-SOMA criteria, and cosmetic outcomes were graded using BCCT.core software.

**Results:**

In 591 patients (median follow-up 5.4 years) RFR was 97.6% (CI: 96.1, 99.2) and 97.1% (CI: 95.2, 98.9) after 5 and 7 years. Overall survival was 96.2% (CI: 94.4, 98.1) and 91.8% (CI: 91.5, 92.1) after 5 and 7 years. We observed either no or mild late toxicities after 7 years. The most frequent toxicities were fibrosis (grade II-III: 15.7%), pain (grade II-III: 3.3%), retractions (grade I: 30%), and teleangiectasia (grade I: 8.9%). Chronic higher-grade fibrosis was seen in 10% and chronic pain in 2% after 7 years in patients treated with IORT only. Cosmetic outcomes were excellent or good for most patients.

**Conclusions:**

The high local control rate and overall survival in combination with low occurrence of late toxicities over 7 years demonstrate that targeted intraoperative radiotherapy is a fast, simple and feasible method during breast-conserving surgery for selected elderly patients.

**Trial registration:**

TARGIT E was prospectively registered at ClinicalTrials.gov with the number NCT01299987 (date: 18 February 2011).

**Supplementary Information:**

The online version contains supplementary material available at 10.1186/s12885-025-15289-0.

## Background

Breast cancer (BC) is the most frequent malignant tumor in women, approximately every third woman (36%) presenting with an oncological malignancy has BC [1]. For locoregional treatment of early BC, breast-conserving surgery (BCS) followed by postoperative radiotherapy (RT) is recommended for most patients [2]. It has been shown that RT, by whole-breast radiotherapy (WBRT) after BCS, results in an absolute reduction in the 10-year risk of any first recurrence by 15.7% and in the 15-year risk of BC-related mortality by 3.8% [3]. Several studies and meta-analyses have demonstrated that additional RT after BCS is the most effective method to reduce local recurrence [4, 5].

BC is increasingly prevalent in elderly patients per se [6], and life expectancy is steadily increasing [7]. However, elderly patients have generally been underrepresented in clinical trials [8]. Yet, the unique characteristics of this patient population, e.g., general condition, comorbidities and comedication, should warrant a closer look. Also, elderly patients may carry cardiac pacemakers that require special attention when applying RT [9]. Radiation of the whole breast can cause radiation toxicities such as fibrosis and edema, and expose organs like heart [10], lung and thyroid [11] to irradiation that might reduce quality of life (QoL) [12, 13]. RT-associated toxicity rates in elderly patients have been reported to be comparable to the general population [14]. Still, several studies have investigated the omission of RT after BCS in elderly patients [8, 15–18]. In these studies, omission of RT in elderly patients was generally associated with worse outcomes, for instance, increased rates of local recurrence with a hazard ratio of > 10 when RT is omitted [15–18] or reduced overall survival [8]. Sometimes RT is omitted for elderly patients due to fear of toxicity, comorbidities, comedication or presence of pacemakers. Additionally, challenging times, like the COVID pandemic, may cause omission of RT in elderly patients to spare overall treatment time and health care resources. A strategy to limit possible toxicity is to use targeted RT to irradiate the breast partially rather than entirely [19]. This approach may be adequate and sufficient for achieving local cancer control using different techniques such as brachytherapy, external beam irradiation or Intraoperative radiotherapy (IORT) [20–22], as several observational studies and randomized clinical trials have demonstrated that more than 90% of recurrent disease occurs within the index quadrant [21, 23–26]. IORT is a special method for targeted RT that administers radiation during BCS while the patient is still under anaesthesia [27, 28]. This means that BC treatment is completed within one procedure [19]. Patients receiving IORT alone reported better QoL, reflected in significantly less general pain, breast and arm symptoms, and better role functioning compared with patients receiving WBRT [29]. No long-term data evaluating IORT in elderly patients are available up to now. TARGIT-E is a prospective, multicentric, and international single-arm phase II trial (NCT01299987) to evaluate the efficacy and safety of a risk-adapted approach for RT consisting of a single dose of IORT in elderly patients (≥ 70 years) with low-risk BC with subsequent WBRT only when risk factors were present in final histopathology. To closely mirror the elderly real-world patient population, TARGIT-E included elderly patients without restrictions regarding the general conditions and comorbidities.

The first results of TARGIT-E with a median follow-up of 3.25 years showed a local recurrence-free rate (RFR) of 99.8% after 2.5 years [30]. Here, we report the results of TARGIT-E after a median follow-up of 5.4 years with a special additional focus on long-term toxicities as well as cosmetic results.

## Methods

The study protocol has been published previously [31]. Patients with low-risk BC (≥ 70 years, cT1-2, cN0, cM0, invasive carcinoma of no special type) were enrolled between February 2011 and September 2014. Patients were enrolled at 28 centers in Europe. The centers in Germany were Berlin, Bottrop, Essen, Hamburg (three centers), Hamm, Homburg, Köln (two centers), Hannover, Ludwigsburg, Magdeburg, Mannheim, München (two centers), Meiningen, Nürnberg, Regensburg and Westerstede. International centers were Frauenfeld (Switzerland), Bordeaux (France), Dijon (France), Lyon (France), Marseille (France), Montpellier (France), Nantes (France), and Herlev (Denmark). All patients signed informed consent to participate in the study. The protocol and all amendments were approved by ethics committees at all participating institutions.

**Fig. 1:**
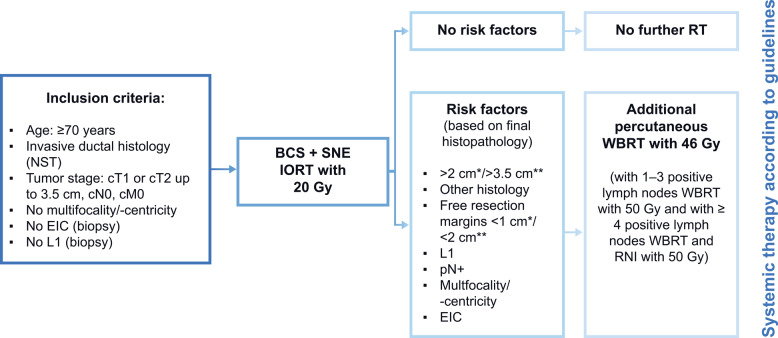
Study design/treatment scheme of TARGIT-E. *In German, Switzerland and Denmark; **In France; In case of positive margins, re-resection should be done. BCS: Breast-conserving surgery; EIC: Extensive intraductal component; IORT: Intraoperative radiotherapy; L1: Lymphangio invasion; NST: No special type; pN + : Positive lymph nodes; RNI: Regional nodal irradiation; RT: Radiotherapy; SNE: Sentinel node biopsy; WBRT: Whole-breast radiotherapy

During breast-conserving surgery, a single dose of 20 Gy low-energy IORT with 50 kV was given (INTRABEAM, Carl Zeiss Meditec, Germany). Additional postoperative WBRT (46–50 Gy) was applied in case risk factors were present in the final histopathology report (see final study design/treatment scheme in Fig. 1). In total, 383 patients received IORT alone (65%), 132 patients received IORT + WBRT (22%) and 36 patients received WBRT alone (6%) due to large tumor cavity or too small skin-applicator distance.

Systemic therapy was applied according to international guidelines [32]. The primary endpoint was the local recurrence (event within 2 cm around the tumor bed) rate according to Kaplan–Meier-estimates after 2.5, 5 and 7.5 years. Further endpoints were overall survival (death from any cause as an event), ipsilateral recurrence (event > 2 cm away from the tumor bed) and contralateral breast cancer (every event in the contralateral breast), lymph node recurrence (any event in the ipsilateral regional lymphatic drainage), distant metastases (any other breast cancer related active tumor not included in the other endpoints), toxicities, and cosmetic results. Toxicities were assessed by a physician using LENT-SOMA criteria [33] during scheduled follow-up timepoints. If patients experienced multiple events, the event relevant to the analyzed endpoint was evaluated. For example, if a patient experienced a local recurrence followed by a metastasis, she was included in the RFR analysis at the time of the local recurrence and in the metastasis analysis at the time of the metastasis. For RFS, either a local recurrence or death was counted as an event, depending on which occurred first. Cumulative rates from freedom of chronic toxicity (occurring at least 3 times during follow-up) were estimated using the Kaplan–Meier method for fibrosis grade II–III, pain grade II–III, any teleangiectasia or retraction. Cosmetic outcomes were analyzed for a subset of patients and rated via BCCT.core as excellent, good, fair, or poor [34]. BCCT.core was assessed preoperatively and after 6 weeks, 3 months, 6 months, and then every year up to 10 years after the intervention. Local recurrence-free rate (RFR) and local recurrence-free survival (RFS) were calculated using the Kaplan–Meier method. Discontinuation of the trial was considered necessary if the local recurrence rates exceeded 3/4/6% at 2.5/5/7.5 years. Absolute and relative frequencies were calculated for qualitative variables and minimum, maximum, median, mean and standard deviation were determined for quantitative variables. To identify a relationship between two qualitative parameters, the chi-square test or, if necessary due to the data, Fisher's exact test was used. Survival curves were generated by the Kaplan–Meier method. Comparison of the Kaplan–Meier curves was performed with the log-rank test. All statistical calculations were performed using the SAS software, release 9.4 (SAS Institute, Inc, Cary, NC). A test result has been assumed statistically significant for a p-value less than 0.05. This is an intention-to-treat analysis.

## Results

For this analysis, 591 patients with a median patient age of 74 years were included. The median follow-up was 65 months (5.4 years. Most patients (65%) received IORT only. 22% received IORT + WBRT and 6% received WBRT only, due to a large tumor cavity or the tumor being very close to the skin. There were 257 right-sided, 327 left-sided, 7 with missing laterality and 301 screen-detected breast cancers. Most included patients had ductal invasive tumor histology. Tumor size was largely T1 with a median tumor size of 12 mm and a range of 1–90 mm. Regarding receptor status, most patients were positive for estrogen receptor (ER) (95%) and positive for progesterone receptor (PR) (56%). Seventeen patients were triple negative. Further selected baseline characteristics are shown in Table 1.

**Table 1:** Baseline characteristics of all patients included in the TARGIT-E study. IORT: Intraoperative radiotherapy; RNI: Regional node irradiation; RT: Radiotherapy; WBRT: Whole-breast radiotherapy

Characteristic	TARGIT-E population (*N* = 591)
Age at enrolment in years, median (min.; max.)	74 (70; 90)
Type of RT, N (%)	
IORT alone	383 (64.8)
WBRT alone	36 (6.1)
IORT + WBRT	132 (22.3)
RNI axilla; supraclavicular fossa	5 (0.8); 3 (0.5)
Not specified	40 (6.8)
Irradiation time (IORT) in minutes, median (min.; max.)	26 (17; 87)
Applicator size in mm, median (min.–max.)	40 (20; 50)
IORT dose in Gy, median	20
WBRT dose in Gy, median (min.–max.)	47 (36—66,4)
Tumor histology, N (%)	
Ductal invasive	542 (91.7)
Infiltrating lobular carcinoma	15 (2.5)
Mixed	11 (1.9)
Not specified	23 (3.9)
Tumor grade, N (%)	
G1	255 (43.1)
G2	266 (45.0)
G3	47 (8.0)
Not specified	23 (3.9)
Tumor size, N (%)	
T1	515 (87.1)
T2	45 (7.6)
T3	1 (0.2)
Not specified	30 (5.1)
Lymph node, N (%)	
Negative	325 (55.0)
Positive	50 (8.5)
Not done	6 (1.0)
Not specified	210 (35.5)
Lymphangio invasion, N (%)	
Yes	39 (6.6)
No	502 (84.9)
Not specified	50 (8.5)
Endocrine therapy, N (%)	
Yes	448 (75.8)
No	128 (21.6)
Not specified	15 (2.5)
Chemotherapy, N (%)	
Yes	36 (6.1)
No	541 (91.5)
Not specified	14 (2.4)

**Fig. 2:**
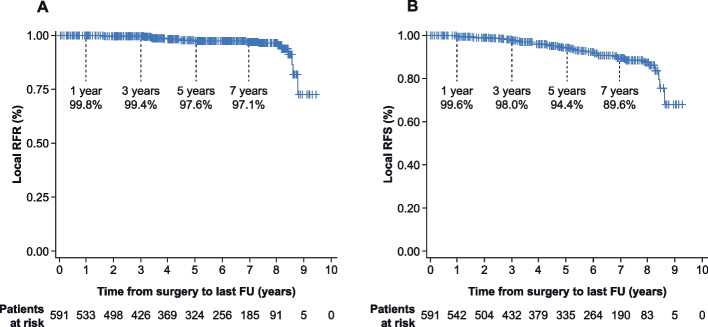
**A** Local recurrence-free rate (RFR). Only local recurrence was counted as an event in this Kaplan–Meier estimate (per protocol). **B** Local recurrence-free survival. Local recurrences and death counted as an event, whatever occurs first. FU: Follow-up, SE: Standard error

Out of 591 patients, 18 (3.0%) had a local recurrence (occurrence 339 to 3141 days after IORT). Local recurrences occurred in 13 out of 383 (3.4%) patients treated with IORT, in 4 out of 132 (3.0%) in patients treated with IORT and WBRT and in 1 out of 36 (2.8%) in patients with WBRT. The local RFR as per protocol was 99.8% (CI: 99.5, 100.0; 1 failure), 99.4% (CI: 98.7, 100.0; 3 failures), 97.6% (CI: 96.1, 99.1; 10 failures), and 97.1% (CI: 95.4, 98.9; 11 failures) of patients free of local recurrence after 1, 3, 5, and 7 years post-treatment, respectively (Fig. 2 A)). Therefore, local recurrence rates of 2.4% at 5 years and 2.9% at 7 years did not exceed the predefined stopping criteria (3/4/6% at 2.5/5/7.5 years).

There were 29 deaths during the follow-up period. Overall survival rates were high (99.8% (CI: 99.4, 100.0; 1 death), 98.3% (CI: 97.2, 99.5; 7 deaths), 96.2% (CI: 94.4, 98.1; 16 deaths), and 91.8% (CI: 91.5, 92.1; 28 deaths) after 1, 3, 5, and 7 years post-treatment). As death is a relevant concurring event in our elderly patient population, we combined local recurrence and death (whatever occurs first) to estimate local recurrence-free survival. Figure 2 B) shows that after 1, 3, 5 and 7 years, 99.6% (CI: 99.1, 100.0; 2 events), 98.0% (CI: 96.7, 99.2; 10 events), 94.4% (CI: 92.1, 96.6; 24 events) and 89.6% (CI: 86.3, 92.9; 37 events) of the patients were alive without local recurrence, respectively.

Regarding other recurrences, ipsilateral recurrence (> 2 cm from the tumor bed) was the most frequent, with 13 recurrences within the study period. Contralateral breast cancer (*N* = 4), locoregional lymph node recurrence (*N* = 5), distant metastasis (*N* = 8), and secondary malignancies (*N* = 3) were also observed (Supplement Table 1).

Late toxicities were assessed by LENT-SOMA criteria [34], ranging from grade 0 (no toxicity) to grade IV (life-threatening toxicity). There were no grade IV or V toxicities during the follow-up period. Toxicities grade II–III were rated here as higher-grade toxicities and considered clinically relevant. For teleangiectasia and retractions, only mild toxicities of grade I were reported but considered clinically relevant for this study. Supplement table 2 overviews all recorded late toxicities according to LENT-SOMA over 7 years of follow-up.overviews all recorded late toxicities according to LENT-SOMA over 7 years of follow-up.

**Fig. 3:**
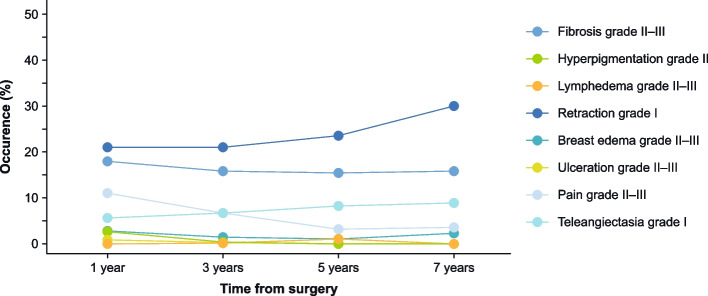
Occurrence of toxicities over time. Depicted are toxicities with LENT-SOMA grade II–III. For retractions and teleangiectasia, grade I is shown, as only mild toxicities of grade I were reported

For most patients and all assessed late toxicities, there were either no toxicities (grade 0) or mild toxicities (grade I). Ulcerations and lymph edemas were very rare in general, with grade II–III toxicities occurring < 1% throughout the follow-up period. Hyperpigmentation grade II only occurred within the first 3 years (2.6% after 1 year, 0.3% after 3 years). The most frequent late toxicities were fibrosis, retractions, pain, and teleangiectasia. Regarding fibrosis, 15–18% of patients exhibited grade II fibrosis and 0.3–1.1% grade III fibrosis throughout the follow-up period; fibrosis rates did not differ substantially after 1, 3, 5, and 7 years. Retractions grade I occurred in 21–30% of patients with no grade II or III retractions through the whole follow-up period. There was a slight trend towards increased mild retraction rates over time. Around 11% of patients reported pain grade II–III after 1 year of follow-up. This rate decreased over time to around 3% after 7 years. Teleangiectasia occurred for approx. 6% of patients after 1 year, with a slight increase in rates over time to 8.9% after 7 years. Figure 3 depicts the time course of late toxicities.

**Fig. 4:**
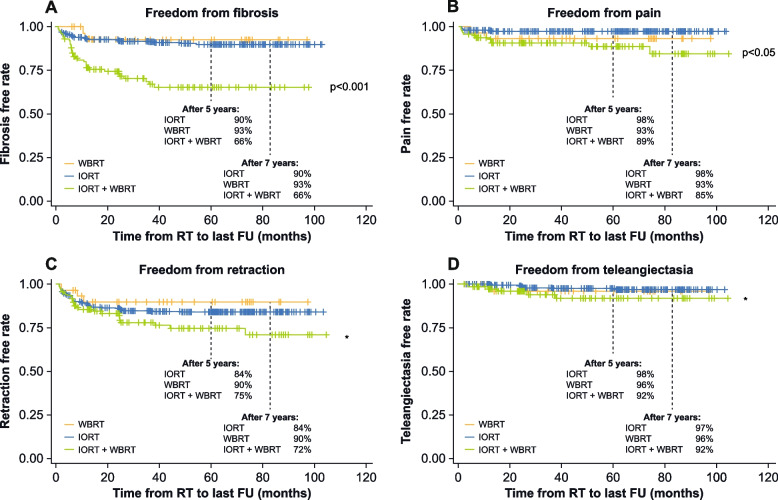
Toxicity-free rates of selected late toxicities, analyzed by the Kaplan–Meier method. **A ** Rate of patients without chronic fibrosis grade II or III; **B ** Rate of patients without chronic pain of the breast grade II or III; **C ** Rate of patients without retraction; **D ** Rate of patients without teleangiectasia. IORT: Intraoperative radiotherapy; WBRT: Whole-breast radiotherapy, *not significant

Freedom from chronic toxicities was analyzed by Kaplan–Meier estimation showing cumulative incidences for the most common toxicities occurring at least three times during follow-up (thereby considered chronic). These analyses also differentiated toxicity-free rates between the different RT modalities: IORT, WBRT, and IORT + WBRT. Figure 4 shows the time course of toxicity-free rates for the four most common late toxicities.

Chronic higher grade fibrosis was seen in 10% and chronic pain grade II or III in 2% after 5 and 7 years in patients treated with IORT only. After 7 years, 97% had no teleangiectasia and 84% no retraction with IORT only. In the case of fibrosis, the group of patients receiving both IORT + WBRT showed significantly lower toxicity-free rates after 5 and 7 years when compared to the groups of patients receiving either IORT or WBRT only (free from fibrosis: IORT after 5 and 7 years: 90%, WBRT after 5 and 7 years: 93% vs. IORT + WBRT after 5 and 7 years: 66%; *p* < 0.001). The same trend was seen for pain (IORT after 5 and 7 years: 98%, WBRT after 5 and 7 years: 93% vs. IORT + WBRT after 5 years: 89% and after 7 years: 85%; *p* < 0.05). For the occurrence of retractions, there was a significant correlation with tumor size (*p* < 0.001).

**Fig. 5:**
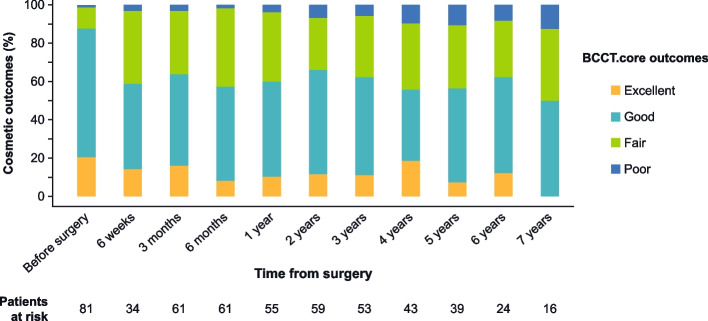
Cosmetic outcomes assessed by BCCT.core over the study period

The subset of available patients with eligible photos for cosmetic analysis were up to 81 patients and is therefore very limited. During follow-up, the number of available patients decreased to 11 patients at 7 years. Because only few patients were available after a follow-up of 7 years no further data are shown for the years 8–10. Preoperatively (*N* = 81), cosmetics were rated as excellent for 21.0% of patients, good for 66.7% of patients, fair for 11.1%, and poor for 1.2% of patients. After the intervention, the percentage of excellent and good cosmetic ratings decreased over the follow-up period (range for excellent outcomes: 0–18.6%; range for good outcomes: 37.2–54.2%). Figure 5 depicts the time course of cosmetic outcomes over the study period.

Cosmetic outcomes were also evaluated with regard to RT modality over the whole study period. There were 54 excellent results in total, 35 of which were with IORT only (64.8%), 6 were with WBRT only (11.1%), and 13 were with the combination of IORT and WBRT (24.1%). Good cosmetic outcomes were reported in 220 patients; of these, 135 were treated with IORT only (61.4%), 30 with WBRT only (13.6%), and 55 with IORT + WBRT (25.0%). Fair cosmetic outcomes occurred in 156 cases, 68 with IORT only (43.6%), 16 with WBRT only (10.3%), and 72 with IORT + WBRT (46.1%). Lastly, there were 26 poor results in total, 11 with IORT only (42.3%) and 15 with IORT + WBRT (57.7%).

## Discussion

This study evaluated risk-adapted intraoperative radiotherapy (IORT) during breast-conserving surgery in patients ≥ 70 years. Most received IORT alone. After > 7 years, recurrence rates were low, with local control and overall survival > 90%. Late toxicities were infrequent and mild, particularly after IORT alone. Cosmetic outcomes were mostly good to excellent, favoring IORT over WBRT. IORT, especially given as a sole treatment, appears to be an effective, well-tolerated, and cosmetically favorable option in elderly patients. The rate of additional WBRT to IORT was 22% in this study due to requirements from the national authorities in Germany to add WBRT as soon as a resection margin less than 1 cm was achieved back in the year 2011. In the following study TARGIT C (NCT02290782) they already stepped back and followed the literature, so 1 mm resection margin as recommended in the national guideline is adequate. This should lower the rate of combined treatment and offer much more one-stop-shop treatments for elderly patients with IORT.

Local control was 3% overall in our cohort. When IORT was given alone a further BCS in combination with WBRT can be done to treat a later local recurrence or ipsilateral breast cancer. Additionally, IORT also seems to reduce non-breast cancer mortality and might have beneficial effects compared to WBRT as shown by Vaidya et al. [35]. Patients treated with IORT showed also a better prognosis than patients treated with WBRT with local recurrence [35]. This is also in line with our findings of low metastasis rates and an excellent overall survival of > 90% after 7 years. On a mechanistic level, IORT may improve antitumor immunity, as it was shown that a single dose of IORT can increase the natural killer cell count in peripheral blood. In contrast, immunosuppressive cells involved in immune escape were not stimulated by IORT [36]. Additionally, IORT seems to modulate the tumor microenvironment by altering cytokine expression in the wound fluid leading to reduced proliferation of mesenchymal stromal cells and chemotactic migration activity [37]. Furthermore, IORT has also been successfully used in elderly patients with cardiac pacemakers, not causing pacemaker malfunction, thereby eliminating the necessity of relocating pacemaker surgeries [38, 39]. IORT offers advantages for patients living far away from treatment centers, as complete treatment with BCS and IORT is finished within a “one-stop-shop” procedure, compared to WBRT, which takes longer [40]. Not only is this convenient for the patients, but completing RT within one procedure can substantially reduce CO_2_ emissions caused by frequent trips to the treatment center to receive WBRT, making IORT a sustainable RT method [41, 42]. Vaidya et al. showed also that IORT is highly cost-effective compared to WBRT and also QALYs were higher after IORT compared to WBRT (8.15 vs. 7.97) based on the results from the TARGIT A study [43].

Other studies that evaluated RT in elderly patients have shown that the complete omission of RT showed inferior local recurrence-free rates compared to adjuvant RT. For example, 5-year local RFR in the PRIME II study and the CALGB 9343 study were similar to the here reported rates (PRIME II: 98.7%, CALGB 9343: 99%) but significantly lower when RT was omitted [15, 16]. Both in the long-term follow-up of PRIME II (median follow-up: 9.1 years) and CALGB 9343 (median follow-up: 12.6 years), the difference in local RFR was even more pronounced and significantly in favor of WBRT after BCS (PRIME II: 90.5% without RT and 99.1% with RT, HR 10.4 (*p* < 0.001) [44]; CALGB 9343: 90% without RT and 98% with RT, HR: 5.56 (*p* < 0.001)) [17]. Looking at survival, the PRIME II study showed equivalent overall survival rates of ~ 80% in both groups, which is 10% lower compared to the TARGIT-E population (~ 90%). This supports data from TARGIT-A and other analysis of cohorts treated with IORT showing consistently high overall survival rates [45, 46]. Another study showed also that WBRT in addition to BCS in elderly patients lead to significantly better local control in a real-life setting [47].

Concerning toxicities, targeted IORT can reduce the irradiation of healthy tissues, decreasing RT-related toxicities [19]. In TARGIT-E, we observed either no late toxicities or mild ones for most patients. Freedom from chronic toxicity was remarkable high after IORT alone and less when IORT was given in combination with WBRT. These rates for the combined treatment of IORT and WBRT are comparable with toxicity rates reported in the TARGIT-BQR phase IV registry study that analyzed toxicity data of 902 patients with a median age of 61 years treated with IORT and WBRT [48]. Retractions might be more frequent in elderly patients as skin elasticity and skin recovery decrease with age [49]. However, we did not find a clear trend, and furthermore, all reported retractions were mild (grade I).

In TARGIT-E, we found a trend towards more excellent and good results for patients that received IORT only compared to WBRT only and IORT + WBRT. These results have, however, to be interpreted with caution, as the total number of evaluated patients (especially at later time points) was low. Additionally, the cause-and-effect relationship is not unambiguous, as patients who received IORT + WBRT generally had additional risk factors such as larger tumor size or multifocality and additional treatment with chemotherapy.

Limitations of this study are the single-arm design and that the assessment of late toxicities and cosmetic results, although performed with standardized measures, is nevertheless based on subjective evaluation by physicians and photographic documentation (with a software-based evaluation). Also, many patients were lost to follow-up over time regarding assessment of late toxicities and cosmetic results, e.g., as participation of patients in follow-up visits in the clinic decreased over time because of logistical effort, pandemic situation (COVID-19) and/or reduced mobility, which might be especially relevant to elderly patients.

## Conclusions

Our results show that targeted IORT is an effective, fast and feasible method for RT during BCS, especially for elderly patients. Over 7 years of follow-up, local RFR and overall survival were very high, and rates of late toxicities assessed by LENT-SOMA were largely non-existent or mild and comparable to toxicity rates for younger BC patients. Cosmetic outcomes were largely excellent or good and also comparable to cosmetic outcomes for younger patients. Therefore, IORT is a very interesting single-shot solution for elderly patients with early breast cancer.

## Supplementary Information

Supplementary Material 1.

Supplementary Material 2.

## Data Availability

The datasets analysed during the current study are available in compliance with data protection regulations (GDPR) from the corresponding author on reasonable request.

## Abbreviations

APBI: Accelerated partial breast irradiation
BC: Breast cancer
BCS: Breast-conserving surgery
IORT: Intraoperative radiotherapy
RFR: Local recurrence-free rate
WBRT: Whole-breast radiotherapy
LENT-SOMA: Late Effects Normal Tissue—Subjective, Objective, Management, Analytic
TARGIT: TARGeted intraoperative radioTherapy
RFS: Recurrence-free survival
QoL: Quality of life

## References

[1] Smolarz B, Nowak AZ, Romanowicz H. Breast cancer—epidemiology, classification, pathogenesis and treatment (review of literature). Cancers (Basel). 2022;14(10):2569. 10.3390/cancers14102569. 10.3390/cancers14102569 PMC9139759 35626173

[2] Loibl S, André F, Bachelot T, Barrios CH, Bergh J, Burstein HJ, et al. Early breast cancer: ESMO clinical practice guideline for diagnosis, treatment and follow-up. Ann Oncol. 2024;35(2):159–82. 38101773 10.1016/j.annonc.2023.11.016

[3] Effect of radiotherapy after breast-conserving surgery on 10-year recurrence and 15-year breast cancer death: meta-analysis of individual patient data for 10 801 women in 17 randomised trials. Lancet. 2011;378(9804):1707–16. 10.1016/S0140-6736(11)61629-2 PMC3254252 22019144

[4] Clarke M, Collins R, Darby S, Davies C, Elphinstone P, Evans V, et al. Effects of radiotherapy and of differences in the extent of surgery for early breast cancer on local recurrence and 15-year survival: an overview of the randomised trials. Lancet. 2005;366(9503):2087–106. 16360786 10.1016/S0140-6736(05)67887-7

[5] Early Breast Cancer Trialists' Collaborative G, Darby S, McGale P, Correa C, Taylor C, Arriagada R, et al. Effect of radiotherapy after breast-conserving surgery on 10-year recurrence and 15-year breast cancer death: meta-analysis of individual patient data for 10,801 women in 17 randomised trials. Lancet. 2011;378(9804):1707–16. 10.1016/S0140-6736(11)61629-2 PMC3254252 22019144

[6] Biganzoli L, Battisti NML, Wildiers H, McCartney A, Colloca G, Kunkler IH, et al. Updated recommendations regarding the management of older patients with breast cancer: a joint paper from the European Society of Breast Cancer Specialists (EUSOMA) and the International Society of Geriatric Oncology (SIOG). Lancet Oncol. 2021;22(7):e327–40. 34000244 10.1016/S1470-2045(20)30741-5

[7] Vollset SE, Ababneh HS, Abate YH, Abbafati C, Abbasgholizadeh R, Abbasian M, et al. Burden of disease scenarios for 204 countries and territories, 2022–2050: a forecasting analysis for the Global Burden of Disease Study 2021. Lancet. 2024;403(10440):2204–56. 38762325 10.1016/S0140-6736(24)00685-8 PMC11121021

[8] Lai X, Han W, Zhang H, Hou J, Wang G, Luo X, et al. Prognostic role of radiotherapy in low-risk elderly breast cancer patients after breast-conserving surgery: a cohort study. Gland Surg. 2022;11(5):847–59. 35694094 10.21037/gs-22-235 PMC9177274

[9] Menard J, Campana F, Kirov KM, Bollet MA, Dendale R, Fournier-Bidoz N, et al. Radiothérapie pour un cancer du sein et stimulateur cardiaque. Cancer/Radiothérapie. 2011;15(3):197–201. 21420890 10.1016/j.canrad.2010.11.014

[10] Zureick AH, Grzywacz VP, Almahariq MF, Silverman BR, Vayntraub A, Chen PY, et al. Dose to the left anterior descending artery correlates with cardiac events after irradiation for breast cancer. Int J Radiat Oncol Biol Phys. 2022;114(1):130–9. 35483540 10.1016/j.ijrobp.2022.04.019

[11] Zhao XR, Fang H, Jing H, Tang Y, Song YW, Liu YP, et al. Radiation-induced hypothyroidism in patients with breast cancer after hypofractionated radiation therapy: a prospective cohort study. Int J Radiat Oncol Biol Phys. 2023;115(1):83–92. 36306978 10.1016/j.ijrobp.2022.04.052

[12] Batenburg MCT, Bartels M, Maarse W, Witkamp A, Verkooijen HM, van den Bongard HJGD. Factors associated with late local radiation toxicity after post-operative breast irradiation. Breast J. 2022;2022:1–13. 10.1155/2022/6745954 PMC9187272 35711897

[13] Hickey BE, Lehman M. Partial breast irradiation versus whole breast radiotherapy for early breast cancer. Cochrane Database of Systematic Reviews. 2021;(8):CD007077. 10.1002/14651858.CD007077.pub4 PMC8406917 34459500

[14] Zamagni A, Buwenge M, Ammendolia I, Ferioli M, Mandrioli A, Morganti AG, et al. Radiotherapy in elderly patients with breast cancer: a literature review of acute and late toxicity. Transl Cancer Res. 2020;9(S1):S173–88. 35117961 10.21037/tcr.2019.08.28 PMC8798880

[15] Kunkler IH, Williams LJ, Jack WJL, Cameron DA, Dixon JM. Breast-conserving surgery with or without irradiation in women aged 65 years or older with early breast cancer (PRIME II): a randomised controlled trial. Lancet Oncol. 2015;16(3):266–73. 25637340 10.1016/S1470-2045(14)71221-5

[16] Hughes KS, Schnaper LA, Berry D, Cirrincione C, McCormick B, Shank B, et al. Lumpectomy plus tamoxifen with or without irradiation in women 70 years of age or older with early breast cancer. N Engl J Med. 2004;351(10):971–7. 15342805 10.1056/NEJMoa040587

[17] Hughes KS, Schnaper LA, Bellon JR, Cirrincione CT, Berry DA, McCormick B, et al. Lumpectomy plus tamoxifen with or without irradiation in women age 70 years or older with early breast cancer: long-term follow-up of CALGB 9343. J Clin Oncol. 2013;31(19):2382–7. 23690420 10.1200/JCO.2012.45.2615 PMC3691356

[18] Tinterri C, Gatzemeier W, Zanini V, Regolo L, Pedrazzoli C, Rondini E, et al. Conservative surgery with and without radiotherapy in elderly patients with early-stage breast cancer: a prospective randomised multicentre trial. Breast. 2009;18(6):373–7. 19910194 10.1016/j.breast.2009.09.013

[19] Vaidya JS, Bulsara M, Baum M, Wenz F, Massarut S, Pigorsch S, et al. Long term survival and local control outcomes from single dose targeted intraoperative radiotherapy during lumpectomy (TARGIT-IORT) for early breast cancer: TARGIT-A randomised clinical trial. BMJ. 2020. 10.1136/bmj.m2836 PMC7500441 32816842

[20] Baum M, Vaidya JS, Mittra I. Multicentricity and recurrence of breast cancer. Lancet. 1997. 10.1016/S0140-6736(05)60950-6. 9111565 10.1016/S0140-6736(05)60950-6

[21] Vaidya JS, Joseph DJ, Tobias JS, Bulsara M, Wenz F, Saunders C, et al. Targeted intraoperative radiotherapy versus whole breast radiotherapy for breast cancer (TARGIT-A trial): an international, prospective, randomised, non-inferiority phase 3 trial. Lancet. 2010;376(9735):91–102. 20570343 10.1016/S0140-6736(10)60837-9

[22] Vaidya JS, Vyas JJ, Chinoy RF, Merchant N, Sharma OP, Mittra I. Multicentricity of breast cancer: whole-organ analysis and clinical implications. Br J Cancer. 1996;74(5):820–4. 8795588 10.1038/bjc.1996.442 PMC2074702

[23] Boyages J, Recht A, Connolly JL, Schnitt SJ, Gelman R, Kooy H, et al. Early breast cancer: predictors of breast recurrence for patients treated with conservative surgery and radiation therapy. Radiother Oncol. 1990;19(1):29–41. 2173044 10.1016/0167-8140(90)90163-q

[24] Clark RM, McCulloch PB, Levine MN, Lipa M, Wilkinson RH, Mahoney LJ, et al. Randomized clinical trial to assess the effectiveness of breast irradiation following lumpectomy and axillary disection for node-negative breast cancer. JNCI J Natl Cancer Inst. 1992;84(9):683–9. 1314910 10.1093/jnci/84.9.683

[25] Veronesi U, Luini A, Del Vecchio M, Greco M, Galimberti V, Merson M, et al. Radiotherapy after breast-preserving surgery in women with localized cancer of the breast. N Engl J Med. 1993;328(22):1587–91. 8387637 10.1056/NEJM199306033282202

[26] Vaidya JS, Tobias JS, Baum M, Keshtgar M, Joseph D, Wenz F, et al. Intraoperative radiotherapy for breast cancer. Lancet Oncol. 2004;5(3):165–73. 15003199 10.1016/S1470-2045(04)01412-3

[27] Vaidya JS, Baum M, Tobias JS, D’Souza DP, Naidu SV, Morgan S, et al. Targeted intra-operative radiotherapy (Targit): an innovative method of treatment for early breast cancer. Ann Oncol. 2001;12(8):1075–80. 11583188 10.1023/a:1011609401132

[28] Vaidya JS, Baum M, Tobias JS, Morgan S, D’Souza D. The novel technique of delivering targeted intraoperative radiotherapy (Targit) for early breast cancer. Eur J Surg Oncol. 2002;28(4):447–54. 12099658 10.1053/ejso.2002.1275

[29] Welzel G, Boch A, Sperk E, Hofmann F, Kraus-Tiefenbacher U, Gerhardt A, et al. Radiation-related quality of life parameters after targeted intraoperative radiotherapy versus whole breast radiotherapy in patients with breast cancer: results from the randomized phase III trial TARGIT-A. Radiat Oncol. 2013. 10.1186/1748-717X-8-9. 23294485 10.1186/1748-717X-8-9 PMC3896671

[30] Wenz F. TARGIT E(lderly): Prospective phase II trial of intraoperative radiotherapy (IORT) in elderly patients with small breast cancer. J Clin Oncol. 2019;37(15_suppl):563-.

[31] Neumaier C, Elena S, Grit W, Yasser A-M, Uta K-T, Anke K, et al. TARGIT-E(lderly)—prospective phase II study of intraoperative radiotherapy (IORT) in elderly patients with small breast cancer. BMC Cancer. 2012. 10.1186/1471-2407-12-171. 22569123 10.1186/1471-2407-12-171 PMC3519765

[32] Burstein HJ, Curigliano G, Thürlimann B, Weber WP, Poortmans P, Regan MM, et al. Customizing local and systemic therapies for women with early breast cancer: the St. Gallen International Consensus Guidelines for treatment of early breast cancer 2021. Ann Oncol. 2021;32(10):1216–35. 34242744 10.1016/j.annonc.2021.06.023 PMC9906308

[33] Pavy JJ, Denekamp J, Letschert J, Littbrand B, Mornex F, Bernier J, et al. Late effects toxicity scoring: the soma scale. Int J Radiat Oncol Biol Phys. 1995;31(5):1043–7. 7713775 10.1016/0360-3016(95)00059-8

[34] Cardoso JS, Cardoso MJ. Towards an intelligent medical system for the aesthetic evaluation of breast cancer conservative treatment. Artif Intell Med. 2007;40(2):115–26. 17420117 10.1016/j.artmed.2007.02.007

[35] Vaidya JS, Bulsara M, Baum M, Wenz F, Massarut S, Pigorsch S, et al. New clinical and biological insights from the international TARGIT-A randomised trial of targeted intraoperative radiotherapy during lumpectomy for breast cancer. Br J Cancer. 2021;125(3):380–9. 34035435 10.1038/s41416-021-01440-8 PMC8329051

[36] Linares-Galiana I, Berenguer-Frances MA, Cañas-Cortés R, Pujol-Canadell M, Comas-Antón S, Martínez E, et al. Changes in peripheral immune cells after intraoperative radiation therapy in low-risk breast cancer. J Radiat Res. 2021;62(1):110–8. 33006364 10.1093/jrr/rraa083 PMC7779348

[37] Wuhrer A, Uhlig S, Tuschy B, Berlit S, Sperk E, Bieback K, et al. Wound fluid from breast cancer patients undergoing intraoperative radiotherapy exhibits an altered cytokine profile and impairs mesenchymal stromal cell function. Cancers (Basel). 2021. 10.3390/cancers13092140. 33946741 10.3390/cancers13092140 PMC8124792

[38] Bhimani F, Johnson K, Brodin NP, Tomé WA, Fox J, Mehta K, et al. Case report: can targeted intraoperative radiotherapy in patients with breast cancer and pacemakers be the new standard of care? Front Oncol. 2022. 10.3389/fonc.2022.927174. 35903710 10.3389/fonc.2022.927174 PMC9315093

[39] Ramdas Y, Benn C-A, Grubnik A, Mayat Y, Holmes DR. Targeted intraoperative radiotherapy is a safe approach for patients with pacemakers: a case study and literature review. Case Rep Oncol. 2020;13(2):916–22. 32884540 10.1159/000508946 PMC7443642

[40] Abdelsattar JM, McClain K, Afridi FG, Wen S, Cai Y, Musgrove KA, et al. Intraoperative radiation therapy versus whole breast radiation for early-stage breast cancer treatment in rural Appalachia. Am Surg. 2020;86(12):1666–71. 32776782 10.1177/0003134820940735 PMC8366587

[41] Bedir A, Grohmann M, Schafer S, Maurer M, Weimann S, Roers J, et al. Sustainability in radiation oncology: opportunities for enhancing patient care and reducing CO(2) emissions in breast cancer radiotherapy at selected German centers. Strahlenther Onkol. 2024. 10.1007/s00066-024-02303-w. 39317752 10.1007/s00066-024-02303-w PMC12283903

[42] Coombs NJ, Coombs JM, Vaidya UJ, Singer J, Bulsara M, Tobias JS, et al. Environmental and social benefits of the targeted intraoperative radiotherapy for breast cancer: data from UK TARGIT-A trial centres and two UK NHS hospitals offering TARGIT IORT. BMJ Open. 2016;6(5):e010703. 27160842 10.1136/bmjopen-2015-010703 PMC4890331

[43] Vaidya A, Vaidya P, Both B, Brew-Graves C, Bulsara M, Vaidya JS. Health economics of targeted intraoperative radiotherapy (TARGIT-IORT) for early breast cancer: a cost-effectiveness analysis in the United Kingdom. BMJ Open. 2017;7(8):e014944. 28819067 10.1136/bmjopen-2016-014944 PMC5724101

[44] Kunkler IH, Williams LJ, Jack WJL, Cameron DA, Dixon JM. Breast-conserving surgery with or without irradiation in early breast cancer. N Engl J Med. 2023;388(7):585–94. 36791159 10.1056/NEJMoa2207586

[45] Sperk E. Bestrahlen oder nicht bestrahlen bei älteren Patientinnen mit Low-Risk-Mammakarzinom? Erkenntnisse aus den 10-Jahres-Daten der PRIME-II-Studie. Strahlenther Onkol. 2023;199(5):520–1. 36971810 10.1007/s00066-023-02071-z PMC10133080

[46] Abo-Madyan Y, Welzel G, Sperk E, Neumaier C, Keller A, Clausen S, et al. Single-center long-term results from the randomized phase-3 TARGIT-A trial comparing intraoperative and whole-breast radiation therapy for early breast cancer. Strahlenther Onkol. 2019;195(7):640–7. 30796496 10.1007/s00066-019-01438-5

[47] Rogowski P, Schönecker S, Konnerth D, Schäfer A, Pazos M, Gaasch A, et al. Adjuvant therapy for elderly breast cancer patients after breast-conserving surgery: outcomes in real world practice. Cancers. 2023. 10.3390/cancers15082334. 37190263 10.3390/cancers15082334 PMC10137115

[48] Goerdt L, Schnaubelt R, Kraus-Tiefenbacher U, Brück V, Bauer L, Dinges S, et al. Acute and long-term toxicity after planned intraoperative boost and whole breast irradiation in high-risk patients with breast cancer—results from the Targeted Intraoperative Radiotherapy Boost Quality Registry (TARGIT BQR). Cancers (Basel). 2024. 10.3390/cancers16112067. 38893184 10.3390/cancers16112067 PMC11171237

[49] Escoffier C, de Rigal J, Rochefort A, Vasselet R, Leveque JL, Agache PG. Age-related mechanical properties of human skin: an in vivo study. J Invest Dermatol. 1989;93(3):353–7. 2768836

