# Supplementary material for: Intraoperative radiotherapy in elderly patients with breast cancer: long-term follow-up results of the prospective phase II trial TARGIT-E
Source: BMC Cancer. 2025 Dec 4;25:1862. doi: 10.1186/s12885-025-15289-0 (PMC12690916; doi:10.1186/s12885-025-15289-0)
Supplement: Supplementary file 1 — Supplementary Material 1. [file 12885_2025_15289_MOESM1_ESM.docx]

**Supplement Table 1**: Incidence of recurrences during the study period.

| **Type of recurrence (total cohort N=591)** | **N** |
| --- | --- |
| Local recurrence, N (%)  [earliest; latest recurrence in days] | 18 (3.0)  [339; 3141] |
| Ipsilateral recurrence, N (%)  [earliest; latest recurrence in days] | 13 (2.2)  [521; 2081] |
| Lymph node recurrence, N (%)  [earliest; latest recurrence in days] | 5 (0.8)  [379; 1793] |
| Contralateral breast cancer, N (%)  [earliest; latest recurrence in days] | 4 (0.7)  [682; 2574] |
| Distant Metastases, N (%)  [earliest; latest recurrence in days] | 8 (1.4)  [25; 2613] |
| Secondary malignancies, N (%)  [earliest; latest recurrence in days] | 3 (0.5)  [1471; 2403] |
