# Supplementary material for: Intraoperative radiotherapy in elderly patients with breast cancer: long-term follow-up results of the prospective phase II trial TARGIT-E
Source: BMC Cancer. 2025 Dec 4;25:1862. doi: 10.1186/s12885-025-15289-0 (PMC12690916; doi:10.1186/s12885-025-15289-0)
Supplement: Supplementary file 2 — Supplementary Material 2. [file 12885_2025_15289_MOESM2_ESM.docx]

**Supplement Table 2:** Late toxicities according to LENT-SOMA criteria.

| **Toxicity** | **Grade** | **After 1 year** | **After 3 years** | **After 5 years** | **After 7 years** |
| --- | --- | --- | --- | --- | --- |
|  |  | N (%) | N (%) | N (%) | N (%) |
| Fibrosis | 0  I  II  III | N=352  187 (53.1)  101 (28.7)  63(17.9)  1 (0.3) | N=283  157 (55.5)  81 (28.6)  43 (15.2)  2 (0.7) | N=248  150 (60.5)  60 (24.2)  37 (14.9)  1 (0.4) | N=89  53 (59.6)  22 (24.7)  13 (14.6)  1 (1.1) |
| Teleangiectasia | 0  I  II  III | N=360  339 (94.2)  21 (5.8)  –  – | N=285  266 (93.3)  19 (6.7)  –  – | N=249  229 (92.0)  20 (8.0)  –  – | N=90  82 (91.1)  8 (8.9)  –  – |
| Breast edema | 0  I  II  III | N=360  327 (90.8)  23 (6.4)  9 (2.5)  1 (0.3) | N=285  269 (94.4)  12 (4.2)  4 (1.4)  – | N=248  240 (96.8)  6 (2.4)  2 (0.8)  – | N=90  86 (95.6)  4 (4.4)  –  – |
| Retraction | 0  I  II  III | N=358  283 (79.1)  75 (20.9)  –  – | N=285  225 (78.9)  60 (21.1)  –  – | N=249  191 (76.7)  58 (23.3)  –  – | N=90  63 (70.0)  27 (30.0)  –  – |
| Ulceration | 0  I  II  III | N=359  357 (99.4)  –  –  2 (0.6) | N=285  285 (100.0)  –  –  – | N=248  245 (98.8)  2 (0.8)  1 (0.4)  – | N=90  90 (100.0)  –  –  – |
| Lymph edema | 0  I  II  III | N=360  352 (97.8)  8 (2.2)  –  – | N=286  280 (97.9)  6 (2.1)  –  – | N=246  238 (96.8)  6 (2.4)  1 (0.4)  1 (0.4) | N=90  88 (97.8)  2 (2.2)  –  – |
| Hyperpigmentation | 0  I  II  III | N=358  303 (84.6)  46 (12.8)  9 (2.6)  – | N=285  269 (94.4)  15 (5.3)  1 (0.3)  – | N=249  242 (97.2)  7 (2.8)  –  – | N=90  86 (95.6)  4 (4.4)  –  – |
| Pain | 0  I  II  III | N=359  263 (73.3)  57 (15.9)  38 (10.6)  1 (0.3) | N=285  218 (76.5)  48 (16.8)  19 (6.7)  – | N=249  196 (78.7)  46 (18.4)  5 (2.0)  2 (0.8) | N=90  70 (77.8)  17 (18.9)  3 (3.3)  – |
